# Stress in an underground empire

**DOI:** 10.1098/rsbl.2022.0012

**Published:** 2022-03-30

**Authors:** Katarina Medger

**Affiliations:** ^1^ Department of Biology, University of Kentucky, Lexington, KY, USA; ^2^ Mammal Research Institute, Department of Zoology and Entomology, University of Pretoria, Pretoria, South Africa

**Keywords:** mole-rats, reproductive skew, stress, glucocorticoids, colony stability, environmental conditions

## Abstract

The naked mole-rat (*Heterocephalus glaber*) and the Damaraland mole-rat (*Fukomys damarensis*) live in large colonies in underground tunnel systems in sub-Saharan Africa. Most members of the colonies are suppressed from reproduction and they are unlikely to reproduce during their lifetime. Only one female and a small number of males reproduce. This extreme cooperative social system has fascinated researchers since the naked mole-rat was first described as eusocial. Despite much research into the mechanisms of social suppression, the exact mechanisms are still unclear. Much evidence points towards high glucocorticoid concentrations caused by agonistic behaviour by the breeding female suppressing reproduction of non-breeders, but laboratory studies have not found any differences in glucocorticoids between breeders and non-breeders. There is, however, considerable evidence from field studies and other social mole-rats that social stress may indeed be an important factor of social suppression in social mole-rats and that those mechanisms are affected by the stability of the colony and environmental conditions. This review aims to provide a summary of the current knowledge of the relationship between environmental conditions, colony stability, glucocorticoids and reproductive suppression in social mole-rat species and suggests some avenues for future research.

The evolution of social organizations and breeding systems in vertebrates has fascinated researchers for several decades and although much has been discovered, many questions remain to be investigated [1]. The reasons why animals choose to live solitary or in large groups are still largely unresolved and Clutton-Brock [1] argues that more well-planned field studies are required to solve some of these questions. One of the most extreme social systems is found in cooperative breeders in which usually only one male and one female produce all the offspring. Other group members, which are typically the offspring of the breeding pair, do not breed and help with foraging, defence and raising of the young [2]. In the most extreme cases, these non-breeding individuals are almost never able to breed during their lifetime. Although the reasons for why non-breeding individuals do not become reproductively active and seek their own breeding opportunities vary between species, reproduction appears to be suppressed through aggression by the breeding individuals in some cooperative breeders. In meerkats (*Suricata suricatta*), for example, dominant females aggressively evict subordinate females that could become a threat to their breeding monopoly resulting in reproductive downregulation and abortions in the evicted females [3]. Evicted females also show increased levels of faecal glucocorticoid metabolites compared to when they are within their group [3]. Glucocorticoids, such as cortisol and corticosterone, are released upon activation of the hypothalamus–pituitary–adrenal (HPA) axis following a stressful situation. Those hormones act on all levels of the hypothalamus–pituitary–gonadal (HPG) axis and can inhibit reproduction and puberty [4,5]. Subordinates, who experience high dominant aggression, are likely to have higher glucocorticoid levels than dominant individuals in social groups. This was indeed found by Sapolsky [6,7] in olive baboons (*Papio anubi*). Yet, the opposite is also often observed and dominant individuals of many social carnivores have higher glucocorticoid concentrations than subordinates [8]. Possibly the most extreme social system is found in the African mole-rats (Bathyergidae) in which both the naked mole-rat (*Heterocephalus glaber*) and the Damaraland mole-rat (*Fukomys damarensis*) have been described as eusocial with a social system similar to that of social insects [9,10]. Most of the colony members in the two species are barred from reproduction for their entire life [9] and high aggression of the breeding female (often called queen) towards subordinates, particularly higher ranking females, is common especially in naked mole-rats [11,12]. This, and the lack of evidence that pheromones play a role in reproductive suppression in the two species [13–15], suggests that reproduction may be suppressed because of elevated glucocorticoid levels in response to stressful interactions with the breeders. However, no differences in glucocorticoids between breeders and non-breeders have so far been found for either species [13,16,17] causing some authors to contest the possibility of glucocorticoid facilitated suppression of reproduction in mole-rats [16]. Nevertheless, there are several indications that stress plays a role in reproductive suppression in social mole-rats and that environmental factors and social stability of the colony may be of importance. The current review provides an overview of some of the mechanisms of reproductive suppression in the two eusocial mole-rat species as well as some other social mole-rats with a focus on the role of breeder mediated stress and glucocorticoids. The main aim is to start a discussion about how natural environmental variations and colony stability affect reproductive suppression in social mole-rat colonies and to propose some avenues for future research.

The main mechanisms of reproductive suppression differ between the two eusocial species and physiological suppression plays a larger role in naked mole-rats [18,19], whereas inbreeding avoidance appears to be more important in Damaraland mole-rats [13]. In naked mole-rats, dominance by the breeding female and to a lesser extent the breeding male is maintained by nose to nose pushes (shoving). Breeding females tend to shove larger and older non-breeding females and upon queen removal, non-breeding females that were becoming reproductively active were shoving and biting other high-ranking individuals [11]. Furthermore, queen removal results in considerable instability within a colony, which is often associated with the death of a number of individuals. Ultimately, a new queen is recruited from within the colony, which is typically one of the highest ranking females [15,17]. As a result of the fighting and considerable colony instability, plasma and urinary cortisol concentrations are also considerably elevated in all colony members until a new queen is established [15,17]. By contrast, no new queen is recruited from within Damaraland mole-rat colonies. When the queen dies, the colony breaks up and animals disperse although this is likely delayed until the next good rain [9]. In captivity, no new breeding female emerges within Damaraland mole-rat colonies when the previous queen is removed and the colony does not produce any young until a new, unrelated male is introduced [20]. Only the introduction of an unrelated male results in reproductive activation in non-breeding females [13] suggesting that inbreeding avoidance is the main driver maintaining the reproductive skew in Damaraland mole-rats. However, upon the introduction of an unfamiliar male into a functioning colony, aggression within the colony intensifies and non-breeding females show increased reproductive activity resulting in take-overs and a rare case of two females breeding within a colony [12]. Inbreeding avoidance is much more efficient in inhibiting reproduction in colonies in which all individuals are closely related seemingly requiring less aggression and reduced reproductive suppression of non-breeding individuals. Indeed, non-breeding females appear to be less reproductively inhibited in Damaraland mole-rats than in naked mole-rat [19,21–24]. Nevertheless, there are many occasions when inbreeding avoidance may not suffice to suppress reproduction in Damaraland mole-rats. Colony relatedness is considerably lower in Damaraland mole-rats than in naked mole-rats, and more importantly, unrelated individuals of both sexes are found within a colony and some offspring's paternity is different to that of the dominant male [25,26]. This suggests that non-breeding individuals frequently come into contact with unrelated opposite-sex conspecifics providing opportunities for non-breeders to mate; therefore, inbreeding avoidance may not be enough to maintain reproductive inhibition of all colony members in Damaraland mole-rats. Aggression may be used by the breeders to suppress the reproduction of non-breeders when inbreeding avoidance is not sufficient and there are some indications that this is the case at least during parts of the year when dispersals are common.

Damaraland and naked mole-rats occur in semi-desert and desert habitats which are characterized by distinct wet and dry seasons. Soil moisture is low during most of the year making it much harder to dig and increasing the costs of digging extensive tunnel systems [27]. Only when the soil is wet are Damaraland mole-rats able to dig extensive burrow systems with long exploratory tunnels. When the soil is dry, they only dig smaller side tunnels and excavated soil is deposited in the tunnels instead of above ground where the soil forms the characteristic mounts [28]. Rainfall and soil moisture also affect the ability of Damaraland mole-rats to leave their natal colony, disperse and join other colonies or form new colonies. High rainfall results in an increase in the number of small colonies and a decrease of large colonies in an area likely due to non-breeding individuals leaving their colonies [29,30]. Interestingly, males disperse more often and over longer distances than females and are more likely to join an established colony whereas females split off from a colony to start a new one [26,29]. Seasonal differences can also be seen in the reproductive activity and hormone concentrations of subordinate individuals. Non-breeding female Damaraland mole-rats show higher reproductive readiness during the wet compared to the dry season [30,31]. Even more interesting is that plasma testosterone concentrations, and with that possibly aggression, are considerably higher in breeding females [32] during the wet than the dry season. In non-breeding females, plasma testosterone concentrations are much lower throughout the year [32]. By contrast, urinary cortisol concentrations of non-breeding females follow a similar pattern to that of testosterone in breeders and are higher in the wet than the dry season [30]. It is intriguing to postulate that this may be the result of higher aggression from the breeding female and may ultimately result in reproductive suppression of non-breeding females remaining in the colony. Nevertheless, other factors such as the increase in tunnelling, foraging and reproduction associated with the wet season could also explain the higher cortisol concentrations [30]. Taken together, these results point towards considerable changes within Damaraland mole-rat colonies with the onset of the rains and suggest that the threat of dispersal and non-related male intruders combined with a slight increase of reproduction in non-breeding females [26] may be met with aggression by the breeding female. A similar seasonal pattern of testosterone concentrations as in Damaraland mole-rats was also found in the seasonally breeding common mole-rat (*Cryptomys hottentotus hottentotus*) [33]. In addition, queens of the common mole-rat are also more aggressive towards non-breeders around the onset of the breeding season [34]. In meerkats, dominant aggression and high glucocorticoid levels of subordinates are closely linked to times when the pregnancy of a subordinate female would be especially detrimental to the dominants' reproductive success [3,35]. Overall, the findings suggest that opportunities for independent reproduction and dispersal, which are influenced by the seasonal environments most social mole-rats inhabit, are met with higher aggression by the breeding female. In this regard, reproductive suppression of non-breeding females may well be mediated by increased glucocorticoid concentrations.

Studies on other social mole-rats also support the idea that social suppression of reproduction is at least partially mediated through stress-related mechanisms. A recent study used hair samples to measure glucocorticoids in the giant mole-rat (*Fukomys mechowii*), another mole-rat species with a social structure similar to that of the Damaraland mole-rat. They found higher hair cortisol levels in non-breeders than breeders [36]. Glucocorticoids accumulate in the hair over a long time and the measured concentrations are much less affected by circadian changes or other fluctuations and give a better idea of the stress an animal experiences over a longer time period than glucocorticoids measured in blood, urine or faeces [37]. Hair may provide a much better substrate to measure changes in response to antagonistic interactions in social mole-rat colonies that are not happening continuously. Most cases of aggression in giant mole-rats are found between the queen and her oldest daughters, which closely mirrors the pattern found in the naked mole-rat [11,36]. Another aspect that is shared by Damaraland mole-rat, giant mole-rat and another highly social mole-rat, Ansell's mole-rat (*Fukomys anselli*) is that breeders have a much longer lifespan than non-breeders. In captivity, breeders of the giant mole-rat and Ansell's mole-rat live about twice as long as non-breeders [36,38]. This could, of course, be an artefact of captivity, but the same was found for the Damaraland mole-rat in a field study [39] and the same study refers to a personal communication, which suggests that breeding naked mole-rats live about four times as long as non-breeders in the wild. Differences in workload do not appear to explain these differences in ageing as both groups contribute similarly to the social group [38]. Breeding females appear to be somewhat shielded from ageing by their reproductive activity and have, for example, lower oxidative damage than non-breeders [40]. In addition, Bens *et al*. [41] identified transcriptome patterns that could contribute to the breeders' long life and good health, and Sahm *et al*. [42] recently found that the HPA axis plays a role in the extended lifespan of the breeders. Nevertheless, high glucocorticoid concentrations could contribute to the low life-expectancy of non-breeders [36] considering the detrimental effects of chronic stress [43]. This may also explain why an aggression and glucocorticoid-mediated suppression of reproduction is not continuously seen in social mole-rat colonies. The negative effect of chronically elevated glucocorticoid concentrations could cause premature death of non-breeders and as such, threaten the survival of the colony. More studies are needed to further elucidate the connection between social and environmental factors, reproductive suppression through antagonistic interaction, high glucocorticoids and low lifespan of non-breeding social mole-rats.

Despite considerable, although largely inferred, evidence suggesting that social stress plays a part in the reproductive suppression of non-breeders in several species of social mole-rats, no such evidence exists for the naked mole-rat. No difference in glucocorticoid concentrations between breeders and non-breeders or associations between queen aggression and glucocorticoid concentrations of non-breeders has been found in naked mole-rats [11,16,17,44]. All of these studies were conducted in laboratory settings where conditions are constant and the environment is similar to what the mole-rats would experience during the dry season. Laboratory settings do not allow the colony to expand their tunnel systems and disperse and no mole-rats enter these tight systems resulting in very stable colonies, which might only be interrupted by experimental manipulation. Similarly, no consistent differences in urinary cortisol concentrations could be found in Damaraland mole-rats under such constant laboratory conditions [13]. Experimental manipulation of naked mole-rat colonies is difficult as introducing new animals or removing the queen often results in the total destabilization of the colony with high levels of aggression, stress and the death of some individuals [15,17]. This makes it difficult to see more subtle differences in stress responses of non-breeding individuals towards dominance behaviours of the queen that could contribute to the inhibition of reproduction within a colony. Somewhat inadvertently, such a small disturbance to the colony stability of naked mole-rats could be achieved by Blecher *et al*. [45] who removed a large number of non-breeding individuals from an otherwise intact colony. In response, plasma cortisol concentrations increased in female non-breeders left in the colony but not in males. This could have been a response to the manipulation itself and not due to higher aggression by the breeding female but those females also showed higher progesterone concentrations, which were similar to those of the females that were removed at the same time suggesting that reproductive inhibition may have been reduced for a short time [45]. Reproductive suppression was later re-established and progesterone and cortisol levels returned to the levels before the disturbance to the colony [45]. In another study, urinary cortisol concentrations were greater in females of higher rank than in those of a lower rank in a colony which contained several large, high-ranking females [17]. This relationship was not found in any of the other colonies used in the study none of which had a large number of high-ranking females [17]. Large, high-ranking females are more likely to threaten the breeding monopoly of the breeding female. The breeding female also had the highest testosterone concentration in this colony and it is probable that her aggression was focused on the females of higher rank resulting in an increase in their urinary cortisol concentrations [17]. The combined results from these studies suggest that disturbances to the stability of a naked mole-rat colony may reduce reproductive suppression resulting in high aggression and high glucocorticoid concentrations as an attempt by the queen to maintain her breeding monopoly. Nevertheless, colony instability by itself is stressful and it will be a challenge for future studies to distinguish between these and queen mediated effects on glucocorticoids and reproductive suppression. Fortunately, further evidence comes from neuroendocrine studies which have examined the mammalian gonadotrophin-inhibitory hormone (GnIH) (also known as RFamide-related peptide-3 (RFRP-3)) system in the two eusocial mole-rats. GnIH is known to inhibit the HPG axis in mammals and it appears to be linked to the negative effects of stress on reproduction (reviewed in [46]). The distribution of immunoreactive GnIH and GnIH mRNA in naked and Damaraland mole-rats provide promising information towards explaining the role of stress in the social suppression of reproduction in the two species (reviewed in [46,47]), but further studies are needed. In addition, studies have highlighted other pathways, neuropeptides and hormones, such as kisspeptin and the kisspeptin–neurokinin B–dynorphin neurons in the arcuate nucleus of the hypothalamus, oxytocin and vasopressin, and prolactin that could play a role in the social suppression of reproduction in at least some social mole-rat species (reviewed in [46,47]).

Although laboratory studies are unavoidable and important to study subterranean species such as mole-rats, more effort is needed to also study them in their natural habitat including long-term studies that span several seasons or years. As can be seen by the studies on Ansell's and giant mole-rat, studies should not only focus on the two eusocial species, but much information on social group living can be gleaned from the large number of other mole-rat species that exhibit an incredible breadth of social systems and which are still largely unknown. Females are the most interesting sex when studying reproductive suppression in social mole-rats as they are the primary target of suppression and reproductive suppression is typically not as strong in males [48–50]. Males are also not affected in the same way as females by changes in the colony [45]. There are, however, some indications that stress affects the delay in puberty of males [51]. Males are an integral part of a colony providing the same work as females which warrants further investigations into their reproductive biology. Much more effort has been put into understanding the neuroendocrinological mechanisms of reproductive inhibition in naked and Damaraland mole-rats. Nevertheless, more studies are needed and manipulations of the social and natural environment of the social groups may add interesting information on the effects of stress on the reproductive axis. Molecular and transcriptomic studies, as was done by Bens *et al*. [41], could provide much more detailed information on glucocorticoid pathways and components of the HPA axis linked to reproductive suppression in social mole-rats. Such studies would not only be interesting to better understand suppression of reproduction in cooperative breeders, but also contribute important information to biomedical research [46]. In addition, differences in the stress axis between breeders and non-breeders could differentially influence their stress reactivity and ultimately survival [52]. No attempt in this regard has been made to this date and such studies could open up more interesting new research opportunities.

In conclusion, the studies and information reviewed here suggest that aggression by the queen and the associated stress experienced by non-breeders plays a role in the inhibition of puberty and reproduction in social mole-rats. The extent of the role of stress-related suppression appears to be dependent on the stability of the colony, which in turn is related to environmental conditions. There are differences in how the reproductive skew is maintained between species with inbreeding avoidance being more important in Damaraland than naked mole-rats, and it is likely that other mechanisms also play a role. Nonetheless, during some times of the year those mechanisms may not prove enough and the queen may have to aggressively suppress non-breeding females from reproducing. Further observations and studies are needed to substantiate this hypothesis.

## Data Availability

This article has no additional data.
